# Synthesis and Anticancer Activity Assay of Novel Chalcone-Sulfonamide Derivatives

**Published:** 2017

**Authors:** Ahmad Pesaran Seiied Bonakdar, Farzane Vafaei, Mahbobeh Farokhpour, Mohammad Hossein Nasr Esfahani, Ahmad Reza Massah

**Affiliations:** a *Department of Chemistry, Shahreza Branch, Islamic Azad University, 86145–311, Shahreza, Iran.*; b *Department of Pharmacy, Shahreza Branch, Islamic Azad University, 86145–311, Shahreza, Iran. *; c *Royan Institute, Isfahan branch.*

**Keywords:** Anticancer activity, Chalcone, Sulfonamide

## Abstract

Today Cancer remains to be one of the most deadly diseases in the world. Due to the potential anticancer activity of the chalcone and sulfonamide moieties, five novel hybrid compounds containing both structures have been designed and synthesized in 3 steps. The synthesized compounds were established on the basis of IR, ^1^H NMR, ^13^C NMR spectral data, and elemental analysis and also they were screened for *in-vitro* anticancer activity on human breast cancer cell line MCF-7. Among them, (E)–2–methoxy–*N*–(4–methoxyphenyl)–5–(3–(4–nitrophenyl) acryloyl) benzene sulfonamide (4) showed the most potent anticancer activity against MCF-7 cell line.

## Introduction

Chalcones, or 1,3–diaryl–2–propen–1–ones; one of the major classes of natural products with widespread distribution in spices, tea, fruits and vegetables have been recently subject of great interest for their pharmacological activities. Chalcones have been reported to posses many useful properties like anticancer (1), antibacterial (2), antifungal (3), anti–inflammatory (4), antimalarial (5), and anti–diabetic activity (6). 

Moreover, sulfonamide derivatives belong to the most important structural classes of drug molecules. Sulfonamides possess many biological activities such as anticancer (7), antibacterial (8), and antimalarial (9). 

**Scheme 1:**
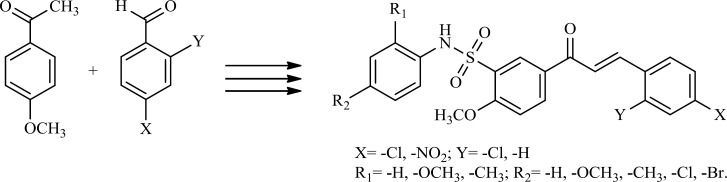
Synthesis of Chalcone sulfonamides

Literature on anticancer chalcones highlights the employment of molecular hybridization through conjugation with other pharmacologically interesting scaffolds for enhancement of anticancer properties. In continuation to extend our research on the synthesis and biological study of sulfonamide derivatives (10, 11), in the present study, we report the synthesis of a series of newly hybrid molecules by combining the structural features of chalcones and sulfonamides (Scheme 1) to test their anticancer activity.

## Experimental

*Materials and measurements*

All chemicals were purchased from Merck and Fluka chemical companies. Melting points were taken in open capillary tubes and are uncorrected. Infrared spectra were recorded on a Perkin–Elmer V IR spectrophotometer. ^1^H NMR and ^13^C NMR spectra were recorded CDCl_3_ on Bruker NMR spectrometers at 400 and 500, 100 and 125 MHz, respectively. Elemental analysis was carried out using Elemental Analyzer for CHN (Perkin Elmer). All reactions were conducted open to the atmosphere and the yields refer to the isolated products.

**Scheme 2:**
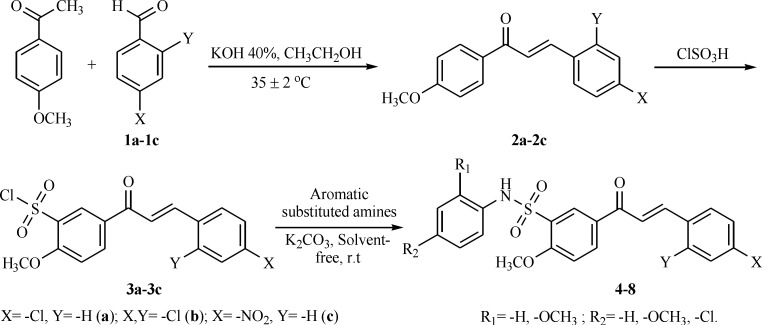
Synthetic route for target compounds 4–8

**Table 1:** Synthesis and anticancer evaluation of new chalcone sulfonamides 4–8

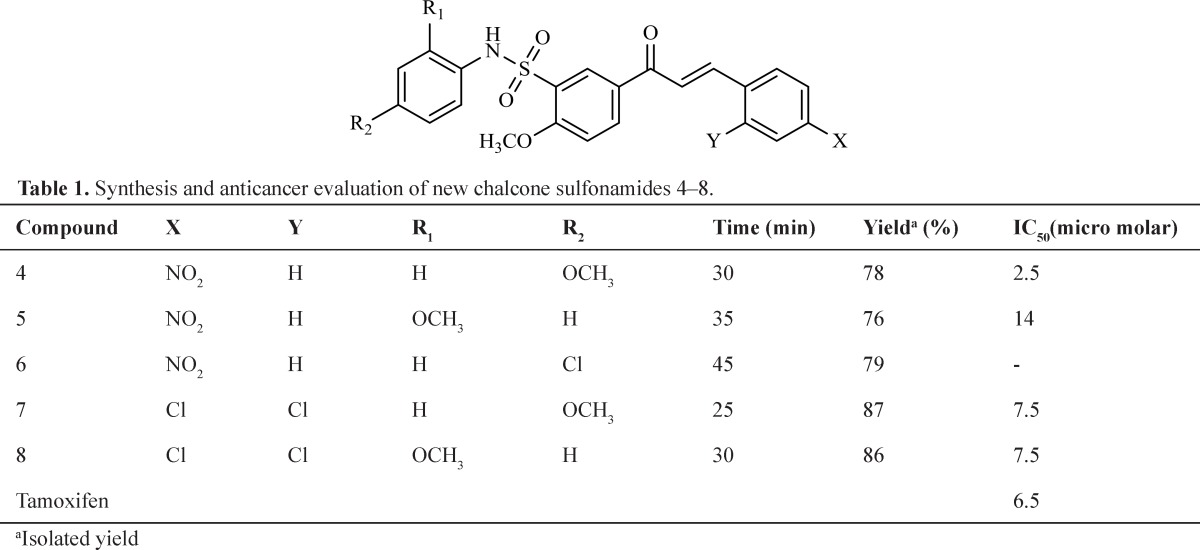

^a^Isolated yield

*Chemistry*

*General procedure for synthesis of chalcone derivatives (2a–2c)*

Chalcones (2a–2c) were prepared by base catalyzed condensation reaction. To a mixture of 4–methoxy acetophenone (13 mmol) and aromatic aldehydes (13 mmol) in ethanol, a solution of potassium hydroxide (40%, 20 mL) were added dropwise over 2–3 min under constant stirring using magnetic stirrer. The reaction mixture was stirred at 35 ± 2 ºC for 4.5–5.0 h. The temperature of the process was maintained at 35 ± 2 ºC by water batch. The progress of the reaction was monitored by TLC, in 70% n–hexane/30% ethyl acetate mixture. After completion of the reaction, the mixture was poured into chilled water (100 mL) and was neutralized with hydrochloric acid. The temperature of the chilled water was in the range of 2–5 ºC. The product was filtered, dried at 60 ºC for 4 h and recrystallized from ethanol.

*General procedure for synthesis of chalcone sulfonyl chloride derivatives (3a–3c)*

Cold chlorosulfonic acid (9 mmol) was added to compound 2a–2c (1 mmol), with stirring for 1h while maintaining the temperature at 0–5 ºC. The reaction mixture was stirred at 35–37 ºC for 1h. The progress of the reaction was monitored by TLC. After completion of the reaction, the mixture was cooled to 0–5 ºC and poured into ice water gradually with vigorous stirring. The crystalline product was separated by filtration.

*General procedure for the synthesis of chalcone sulfonamide derivatives (4–8)*

An amine (1 mmol) and anhydrous K_2_CO_3_ (4 mmol) were ground together into fine powder in a mortar and chalcone sulfonyl chloride derivatives (1 mmol) was added under vigorous stirring with a magnetic stirrer at room temperature. The progress of the reaction was monitored by TLC until the conversion of amine was completed. In all cases the crude products showed one spot on TLC. Upon completion of the reaction, water was added and the mixture was stirred for a few minutes. The precipitate was collected by filtration, washed with water, dried and crystallized from ethanol.

*Anticancer activity*

All the target compounds 4-8 were evaluated for their anticancer activity against MCF-7 cells by MTS assay; DMSO was used as a vehicle. For comparison of the anticancer activity, Tamoxifen was used as the reference agent.

## Results and Discussion

*Chemistry*

The synthesis of chalcone sulfonamide derivatives 4–8 followed the general pathway outlined in Scheme 2. Firstly, the chalcones were synthesized by Claisen–Schmidt condensation reaction of aromatic aldehydes with 4–methoxy acetophenone (1:1), using 40% potassium hydroxide as a base in ethanol. Then, compounds 2a–2c reacted with chlorosulfonic acid at 0–5 ºC to give the corresponding sulfonyl chlorides. Finally, the reaction of chalcone sulfonyl chlorides (3a–3c) with aromatic substituted amines in the presence of K_2_CO_3_ produced chalcone sulfonamides in 79-89% yield under solvent–free conditions (12).

Our results indicate that in general, the synthesis of chalcone sulfonamide derivatives from the corresponding sulfonyl chlorides proceeds in the absence of solvent, with good yields and high purity in relatively short reaction times and according to the principles of green chemistry (Tables 1).

*In-vitro anticancer activity*

The results of anticancer evaluation after 3 days are summarized in Table 1. A careful study revealed that most of the tested compounds showed anticancer activity less than Tamoxifen except compound 4. This chalcone sulfonamides containing 4–nitrophenyl and aryl sulfonamide group substituted with 4-methoxy (4) showed better anticancer activity than others. Compound 3 showed no anticancer activity.

## Conclusion

In conclusion, we synthesized a number of novel chalcone sulfonamide derivatives by the reaction of chalcone sulfonyl chlorides with different aromatic substituted amines in the presence of K_2_CO_3_ under solvent–free conditions at room temperature. All the newly synthesized compounds were assayed for their *in-vitro* anticancer activity against MCF-7. Data obtained indicated that compound **4** exhibited the most potent anticancer activity. More research in this area is in progress.
